# Calcium peroxide alleviates the waterlogging stress of rapeseed by improving root growth status in a rice-rape rotation field

**DOI:** 10.3389/fpls.2022.1048227

**Published:** 2022-11-18

**Authors:** Zhiyuan Wang, Yongliang Han, Shang Luo, Xiangmin Rong, Haixing Song, Na Jiang, Changwei Li, Lan Yang

**Affiliations:** ^1^ College of Resources and Environmental, Hunan Agricultural University, Changsha, China; ^2^ National Engineering Research Center for Efficient Utilization of Soil and Fertilizer Resources, Changsha, China

**Keywords:** waterlogging stress, rapeseed, calcium peroxide, SPAD, antioxidant enzymes, fermentation

## Abstract

Waterlogging stress has a negative influence on agricultural production, particularly for rapeseed yield in a rice-rape rotation field. To alleviate the profound impacts of waterlogging stress on rapeseed production, a new fertilization with calcium peroxide (CaO_2_) was proposed. In this field experiment, with the conventional rape (*Brassica napus* L.) variety fengyou958 (FY958) and early maturing rape variety xiangyou420 (XY420) as materials, waterlogging was imposed from the bud to flowering stage, and three supplies of CaO_2_ (0, C1 for the 594 kg hm^-2^ and C2 for the 864 kg hm^-2^) were added as basal fertilizer. The results showed that CaO_2_ significantly reduced the accumulation of fermentation products in roots and alleviated the peroxidation of leaves. The reduced waterlogging stress promoted the root vigor and agronomic characters, such as branches, plant height and stem diameter, accelerated dry matter and nutrients accumulation, and resulting in 22.7% (C1) to 232.8% (C2) higher grain yields in XY420, and 112.4% (C1) to 291.8% (C2) higher grain yields in FY958, respectively. In conclusion, 594 kg hm^-2^ to 864 kg hm^-2^ CaO_2_ application restored the growth of waterlogged rapeseed leaves, and reduced the anaerobic intensity of root, which enhanced the resistance of plants to waterlogging, and improved crop productivity. In a certain range, the higher CaO_2_ application, the more the yield. This study provides a valid method to prevent damage from flooding in crop fields.

## Highlights

Calcium peroxide alleviated the peroxidation of leaves, altered hormonal activity and reduced the anaerobic intensity of root, which enhanced the resistance of *Brassica napus* L. plants to waterlogging stress.

## Introduction

As one of the most important oil crops, rapeseed (*Brassica napus* L.) ranks first in both yield and planting area in the world. The cultivation of rape in the Yangtze River basin area comprised approximately 85% of the total area of rape cultivated in China (Hu et al., 2017). A rice-rape rotation is the main cropping system in this area. The humidity in the field is excessive, and the soil is clay and heavy during the period of rice growth. High rainfall, poor soil drainage and a high groundwater table combined with a humid climate make rape susceptible to waterlogging. Studies have shown that waterlogging decreases the yield of rapeseed by 17% to 42.4% (Zhou and Lin, 1995; Zou et al., 2014; Hu et al., 2017), and leads to a significant decline in crop quality, resulting in serious economic losses (Dennis et al., 2000; Setter and Waters, 2003).

The deficiency in oxygen and increase in carbon dioxide in the roots of plants grown under waterlogging conditions limits their aerobic functions and enhances their anaerobic metabolism, such as fermentation. As a result, a large quantity of toxic acetic acid, ethanol and acetaldehyde are produced, which change the metabolism of plants and endanger its growth and development (Da-Silva and Amarante, 2020). Moreover, the strengthened anaerobic metabolism will also consume many compounds that are stored in the crop, resulting in poor growth, reduced yield, and even death (Da-Silva and Amarante, 2020). In addition, tolerance to waterlogging changes the validity of nutrients in the soil solution and then affects the absorption, utilization, conversion and redistribution of mineral nutrients in crops (Mei et al., 2017; Men et al., 2020). Under waterlogging stress, the crop roots produce a substantial amount of cortical ventilation tissue, which results in a substantial change in the distribution of oxygen between crop cells, and the cells substantially increased their proportion of aerobic gas exchange (Da-Silva and Amarante, 2020). As a result, the intracellular reductive toxic substances were oxidized, which can normalize the growth and development of the plant (Da-Silva and Amarante, 2020).

One of the best indications of the response of a plant to waterlogging stress is the shift in metabolism from aerobic to anaerobic. Glycolysis and alcohol fermentation are important ways for seeds and seedlings to produce energy under waterlogging conditions. Alcohol dehydrogenase and pyruvate decarboxylase are two important enzymes in the process of anaerobic respiratory alcohol fermentation and play a vital role in the acceleration of plant growth and survival in the absence of oxygen (Shabala et al., 2015; Mei et al., 2017). Hypoxic-resistance plants can maintain an active fermentative metabolism over a long period of time to accelerate the consumption of sugar to increase yield and the sugar metabolic flux two- to three-fold compared with aerobic conditions (Gibbs et al., 2000). The activities of pyruvate decarboxylase and alcohol dehydrogenase decreased five- to ten-fold under aerobic conditions compared with anaerobic conditions (Mustroph et al., 2006).

As stated above, waterlogging is extremely harmful to crops, and it can happen in all stages of plant growth. Thus, it is important to prevent and control waterlogging during production. Calcium peroxide (CaO_2_) is an environmentally friendly material that releases oxygen in the presence of water and is widely used. For the sewage treatment, in the presence of CaO_2_, the growth of M. aerusinosa was severely inhibited and the chlorophyll-a concentration was drastically decreased, while dissolved phosphate in water can be well removed (Lee and Cho, 2002), which was also beneficial to alleviate eutrophication of water bodies. In crop cultivation, pelleting treatments with CaO_2_ significantly increased seed germination and seedling growth of direct-seeded rice under waterlogging conditions (Mei et al., 2017). Meanwhile, CaO_2_ is suitable choice for contaminant biodegradation in soil and ground water, and synthesis of CaO_2_ in nano size by increased ratio of surface to volume can increase the speed of oxidation reaction between CaO_2_ and contaminant (Khodaveisi et al., 2011). However, little work has been done regarding the physiological response of different rapeseed varieties to CaO_2_ and the effect of CaO_2_ on the yield and agronomic characters of rapeseed under waterlogging condition. In this study, with different levels of CaO_2_, the objectives of this work were (1) to examine the effects of different supplies of CaO_2_ on the yield of two rapeseed hybrids, Fengyou 958 (FY958) and Xiangyou 420 (XY420), agronomic traits performance and nutrients accumulation under waterlogging conditions during the bud to flowering stages and (2) to unravel the physiological and root ecological changes in rapeseed plant under the influence of CaO_2_ and waterlogging stress, to obtain a better understanding waterlogging mitigation by CaO_2_ mechanism, which are highly significant to the resistance of rape to flooding while in production.

## Materials and methods

### Field experiment design

The field experiment was conducted from October 2020 to May 2021 in Shuangjiangkou Town, Ningxiang City, Hunan Province, China (28°19′51′′N, 112°38′58′′E). The seeds of Fengyou958 (FY958), a conventional rape variety, and Xiangyou420 (XY420), an early maturing variety of rape, were obtained from the Hunan National Oil Improvement Center in Changsha, China.

The field plot experiment was designed in a random block design with three replicates, and each plot was separated by a ditch. Each plot was 10 m^2^ (6.25 m long x 1.60 m wide). The experimental field was clay soil, with a pH of 5.90 contained 28.57 g kg^-1^ organic matter, 1.35 g kg^-1^ total N, 0.39 g kg^-1^ total P, 12.83 g kg^-1^ total K, 86.42 mg kg^-1^ available N, 19.80 mg kg^-1^ Olsen-P, and 106.67 mg kg^-1^ available K.

The rapeseed was sown on October 29, 2020. Before sowing, base fertilizer was applied, containing N 180 kg hm^-2^, P_2_O_5_ 90 kg hm^-2^, K_2_O 117 kg hm^-2^, and B 3 kg hm^-2^, respectively. Mixed the base fertilizer with CaO_2_, apply it to the soil at one time, and then turn over the soil for covering. The rapeseeds were direct-planted in four rows with a 40 cm space between the plants and 500 rapeseed seeds per plot. 115 days after sowing, four treatments were applied to the plants: (i) WD, well-draining, (ii) C0+WL (waterlogging), no CaO_2_ and waterlogging, (iii) C1+WL, 594 kg hm^-2^ of CaO_2_ in the form of base fertilizer and waterlogging, and (iv) C2+WL, the same as treatment (iii), but the supply of CaO_2_ was 864 kg hm^-2^. The rapeseed waterlogged on February 21, 2021, and the water level was maintained 1 cm above the soil surface for 30 days to create waterlogging conditions. Weed growth in plots was controlled by herbicides before seedling emergence. Plots were kept free of insects and diseases by using fungicide and pesticide when necessary.

### Plant sampling and the determination of N, P, and K

At the end of the waterlogging period and the physiological maturity, five uniform and representative rapeseed plants were sampled from each plot. After a determination of the agronomic traits, the plants were neatly cut with scissors at the stem close to the surface of soil. The leaf blade SPAD was measured from an average of 10 plants per pot before they were sampled. The plants were measured at 10:00 a.m. using a SPAD-502 Plus chlorophyll meter (Konica Minolta Optics, Tokyo, Japan). The leaves and roots were then quickly frozen with liquid nitrogen and stored at -80°C for physiological testing. The plants were harvested at physiological maturity to determine their yield. The leaves, stems and pods were separated from the plants and dried for 30 min in a 105°C oven and then dried to a constant weight at 70°Cand weighed to determine the accumulation of dry matter. After the plants had been ground into fine powder by a grinding miller, the nitrogen (N), phosphorus (P) and potassium (K) content of the plants were determined. N was determined by the Kjeldahl nitrogen method (Chen et al., 2014). P was determined by vanadium molybdate yellow colorimetry, and K was determined using flame photometry (Nielsen, 2017). The Harvest index (HI) was calculated based on a single plant as previously described (Chen et al., 2015): HI = Grain yield/Total plant DM at maturity.

### Activities of antioxidant and fermentation enzymes

The methods of enzyme extraction were modified by Sivasankar and Oaks (1995). A total of 0.1 g of fresh plant tissue samples were fully ground in liquid nitrogen, and 1 mL of extract (80% methanol) was added. The homogenate was centrifuged for 20 minutes at 16000 g at 4°C, and the supernatant was used as the enzyme extract. The activity of superoxide dismutase (SOD) in the leaves was analyzed as described by Wang and Huang (2015), and the activity of catalase (CAT) was assayed as previously described using the hydrogen peroxide reduction method (Sivasankar and Oaks, 1995). The activity of lactate dehydrogenase (LDH) in the roots was assayed as described by Hanson and Jacobsen (1984a), and the activities of pyruvate decarboxylase (PDC) and alcohol dehydrogenase (ADH) in the roots were assayed as described by Hanson et al. (1984b). The enzymes were assayed using ELISA kits (Shanghai Zcibio Biotech Co., Ltd., Shanghai, China).

### Root lactate, ethanol, and root activity

Lactate and ethanol were extracted as described by Kolb and Joly (2009). A total of 0.1 g of plant tissue samples were fully ground in liquid nitrogen, and 1 mL of 80% methanol was added. The homogenate was centrifuged for 20 min at 8000 g at 4°C, and the contents of lactic acid and ethanol supernatant was measured using ELISA kits from Shanghai Zcibio Biotech Co., Ltd. The root activity was determined by the triphenyl tetrazolium chloride (TTC) method (Zhang et al., 2013) using a kit from Shanghai Zcibio Biotech Co., Ltd.

### Plant hormone contents

The contents of abscisic acid (ABA) and gibberellin (GA) in the leaves were determined using liquid chromatography tandem mass spectrometry (LC-MS/MS) as described by Espasandin et al. (2014) with minor modifications. A total of 0.1 g of fresh leaf samples were fully ground in liquid nitrogen, and 5 mL of 80% methanol was added. The homogenate was centrifuged for 10 min at 5000 g at 4°C. The supernatant was treated with a commercial kit (Shanghai Zcibio Biotechnology Co., Ltd.), and the contents of ABA and GA were determined using a LCMS-8030 (Shimadzu, Tokyo, Japan). The levels of ethylene (ETH) were measured as described by Cheng and Lur (1996) with minor modifications. A total of 0.1g of plant leaf samples were transferred to a brown bottle that contained paper that had been wetted with distilled water. The bottle was sealed with a rubber stopper and incubated for 7 h in the dark at room temperature. A 1.0 mL gas sample was withdrawn from the headspace, and the ethylene level was assayed using an American GC-600 gas chromatograph equipped with a flame ionization detector and an Alumina column.

### Soil properties

At the end of the waterlogging period, five portions of soil were randomly sampled in each plot using the five-spot sampling method (Carter and Gregorich, 2007). Fresh soil samples were stored at -20°C. The soil bulk density (g/cm^3^) and porosity (%) were estimated as described by Al-Shammary et al. (2018). A 100 cm^3^ ring knife was inserted into the soil vertically. The surrounding soil was removed and cut under the ring knife. The soil surface was flattened at both ends of the ring knife, covered, dried to a constant wight in an oven at 37°C and recorded. The empty ring cutter was weighted to calculate the soil bulk density and porosity. The activity of soil catalase (S-CAT) was assayed as described by Stepniewska et al. (2009) using a kit from Shanghai Zcibio Biotech Co., Ltd.

### Statistical analysis

The experimental data were compiled and plotted with Microsoft Excel 2021 (Redmond, WA, USA), analyzed using a one-way analysis of variance (ANOVA) and correlated with SPSS 20.0 (IBM, Inc., Armonk, NY, USA). Multiple comparisons were performed using Duncan’s method.

## Results

### Plant growth, rapeseed yield and nutrient absorption

**Figure 1:**
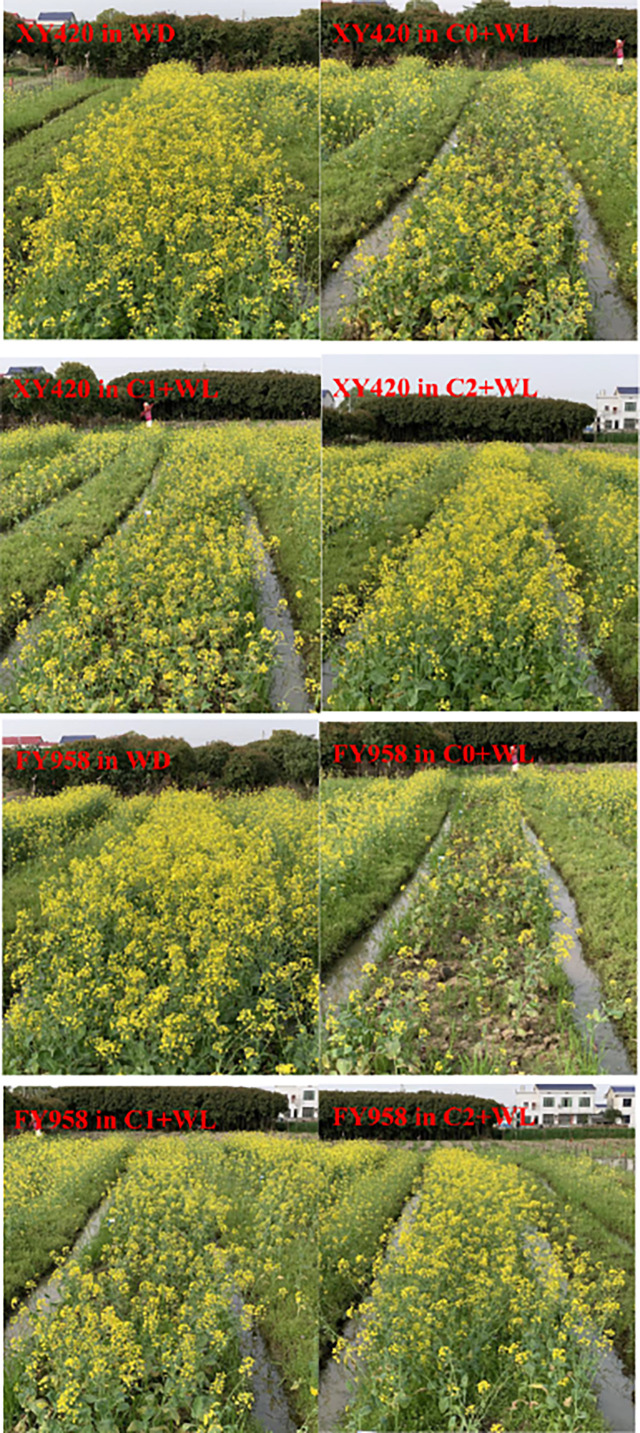
Growth phenotype of rape under different treatments at the end of waterlogging. XY420 in WD, well-draining; XY420 in C0+WL, XY420 with no CaO_2_ and waterlogging; XY420 in C1+WL, XY420 with 594 kg hm^-2^ of CaO_2_ in the form of base fertilizer and waterlogging; XY420 in C2+WL, XY420 with 864 kg hm^-2^ of CaO_2_ in the form of base fertilizer and waterlogging; FY958 in WD, well-draining; FY958 in C0+WL, FY958 with no CaO_2_ and waterlogging; FY958 in C1+WL, FY958 with 594 kg hm^-2^ of CaO_2_ in the form of base fertilizer and waterlogging; FY958 in C2+WL, FY958 with 864 kg hm^-2^ of CaO_2_ in the form of base fertilizer and waterlogging.

The plants were subjected to waterlogging stress from bud to the flowering stage. As shown in **Figure 1**, both varieties of rape had been severely affected by the waterlogging treatments, with a lower plant height, fewer branches and a lower survival density. In addition, the conventional variety FY958 was more sensitive to water stress. However, rape supplied with CaO_2_ (particularly the C2 treatment) grew well even under waterlogging stress (**Figure 1**).

**Figure 2:**
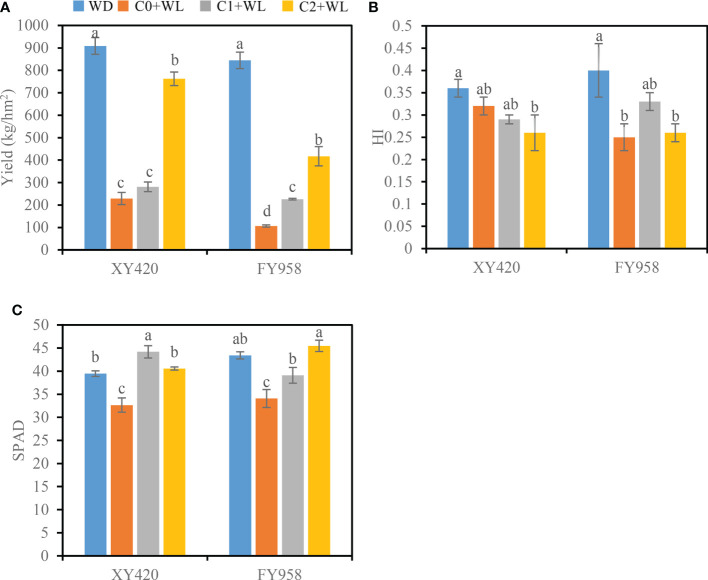
Effects of CaO_2_ on the yield of rape and harvest index (HI) at the harvest stage and leaf SPAD values under waterlogging conditions. **(A)**, yield. **(B)**, HI, harvest index; **(C)**, SPAD, SPAD values. Error bars represent standard errors. Different letters above bars indicate significant differences between planting patterns at *P* < 0.05. WD, well-draining, C0+WL, no CaO_2_ and waterlogging, C1+WL, 594 kg hm^-2^ of CaO_2_ in the form of base fertilizer and waterlogging, and C2+WL as in treatment C1+WL but a supply of 864 kg hm^-2^.

The yield of rapeseed at physiological maturity decreased by 74.8% and 87.4% under waterlogging stress compared with the control, and the HI decreased by 27.8% and 37.5% for XY420 and FY958, respectively (**Figures 2A, B**). Moreover, the pods and seeds of two different rape varieties were significantly reduced after waterlogging and decreased by 63.4% and 15.3%, 69.3% and 23.8% for XY420 and FY958, respectively (**Table 1**). The branches were significantly reduced by 37.5%-81.5% and 30.9%-82.7%, respectively, with a more significant impact on the secondary branches. The diameters of the height and stem decreased significantly by 32.1% and 30.4%, 24.6% and 25.7%, respectively (**Table 1**). However, CaO_2_ application increased the rapeseed yield greatly. For the C1+WL treatments, the yield of rapeseed increased by 22.7 and 112.4% for XY420 and FY958, respectively. For the C2+WL treatments, the yield significant increase of 2.3 and 2.9 times for these two hybrids, respectively (**Figure 2**). CaO_2_ application alleviated the damage of waterlogging stress on agronomic traits. The primary branches were increased by 47.5% and 26.1% for XY420 and FY958 under C1 application, while the secondary branches were increased by 2.5 and 3.1 times under C2 treatment, respectively (**Table 1**). Meanwhile, CaO_2_ makes the plant grow stronger, because plant heights increase by 21.6% and 19.6% under C1 treatment, and 34.0% and 38.4% under C2 treatment for XY420 and FY958. Furthermore, rape stem diameter had risen markedly with CaO_2_ application. For these two hybrids, there was no significant difference between the pods, seeds, branches and heights of the control and C2+WL plants (**Table 1**).

**Table 1:** Effects of calcium peroxide on the agronomic characters of rape at the harvest stage under waterlogging conditions.

Cultivars	Treatments	Pods/plant	Seed/pod	Primary branches	Secondary branches	Height(cm)	Stem diameter(cm)
XY420	WD	298.7 ± 49.4a	26.15 ± 0.46a	6.40 ± 0.59a	6.50 ± 0.30a	121.2 ± 3.4a	3.62 ± 0.18a
	C0+WL	109.9 ± 12.8b	22.10 ± 0.60b	4.00 ± 0.48b	1.20 ± 0.34c	82.3 ± 4.5c	2.73 ± 0.12b
	C1+WL	226.4 ± 23.5a	24.38 ± 1.41ab	5.90 ± 0.70a	4.25 ± 0.57b	100.1 ± 5.1b	3.84 ± 0.21a
	C2+WL	226.8 ± 5.8a	26.00 ± 0.88a	6.25 ± 0.33a	5.05 ± 0.72ab	110.2 ± 0.9ab	3.60 ± 0.08a
FY958	WD	339.9 ± 53.3a	26.90 ± 1.71a	8.33 ± 0.58a	6.35 ± 0.69ab	133.5 ± 8.8a	4.12 ± 0.28ab
	C0+WL	104.5 ± 30.6b	20.50 ± 1.17b	5.75 ± 0.56b	1.10 ± 0.24c	92.9 ± 4.1c	3.06 ± 0.17c
	C1+WL	225.2 ± 21.7a	22.65 ± 0.76b	7.25 ± 0.51ab	4.53 ± 0.64b	111.2 ± 6.6bc	3.63 ± 0.05bc
	C2+WL	223.7 ± 29.8a	28.05 ± 0.69a	7.73 ± 0.64a	7.70 ± 0.50a	128.7 ± 3.7ab	4.36 ± 0.26a

The standard error and significant difference analysis in the table are based on the same cultivar. Data are shown as mean ± SD (n = 4) of four independent experiments. Different letters after the number indicated that there were significant differences among different treatments (P < 0.05). WD, well-draining, C0+WL, no CaO_2_ and waterlogging, C1+WL, 594 kg hm^-2^ of CaO_2_ in the form of base fertilizer and waterlogging, and C2+WL as in treatment C1+WL but a concentration of 864 kg hm^-2^.

Waterlogging during the bud-flowering period can significantly reduce the accumulation of dry matter (DM) in all parts of the harvested rape, and the total plant DM decreased by 69.8% and 70.2% for XY420 and FY958, respectively (**Table 2**). The application of CaO_2_ significantly improved the plant DM by 94.2% and 111.0% in C1 treatment, and by 148.9% and 146.6% in C2 application, for these two hybrids, respectively. The conventional variety FY958 was more sensitive to waterlogging compared with the early maturing variety XY420 owing to its severe decrease in grains. However, CaO_2_ had a more intensive effect on the recovery of growth of XY420. The absorption of N, P and K was also significantly reduced by waterlogging stress compared with the control, which led to a reduction of 64.9%~69.5%, 67.8%~76.1%, and 97.0%~98.9% in the stems, respectively. The contents of N and P in the pods decreased significantly by 50.4%~ 57.8%, and the content of K decreased by 64.6%~71.5%. The contents of total N and P decreased significantly by 57.5%~64.3%, and the contents of total K decreased significantly by 78.3%~84.2%. The application of CaO_2_ significantly improved the absorption of nutrients by both varieties of rape compared with the waterlogging treatment. CaO_2_ increased the accumulation of nutrient elements in various organs and resulted in 71.5% and 82.6% increase in plant N, 95.7% and 86.7% increase in P, 79.8% and 137.4% increase in K under C1 treatment, while led to 121.4% and 92.9% increase in plant N, 73.0% and 170.0% increase in P, 269.9% and 389.1% increase in K under C2 treatment. Higher CaO_2_, better effect, and there was no significant difference between C2 treatment and the control (**Table 3**).

**Table 2:** Effects of calcium peroxide on rape dry matter at the harvest stage under waterlogging conditions.

Cultivars	Treatments	Stem (g/plant)	Fruit pod (g/plant)	Grains (g/plant)	Total (g/plant)
XY420	WD	13.62 ± 0.45a	17.06 ± 1.71a	17.61 ± 1.80a	48.29 ± 3.48a
	C0+WL	3.57 ± 0.26d	6.25 ± 0.84c	4.56 ± 0.51c	14.38 ± 0.91d
	C1+WL	8.78 ± 0.59c	11.22 ± 0.4b	7.93 ± 0.21bc	27.93 ± 0.52c
	C2+WL	10.30 ± 0.26b	16.10 ± 0.9a	9.39 ± 1.5b	35.79 ± 0.95b
FY958	WD	14.08 ± 2.81a	21.33 ± 2.99a	22.75 ± 2.25a	58.15 ± 4.05a
	C0+WL	6.69 ± 1.24b	6.26 ± 0.97c	4.61 ± 1.14c	17.57 ± 2.23c
	C1+WL	13.25 ± 0.71a	11.62 ± 0.89bc	12.19 ± 0.77b	37.07 ± 0.81b
	C2+WL	14.07 ± 1.00a	17.87 ± 2.13ab	11.38 ± 1.39b	43.32 ± 2.62b

The standard error and significant difference analysis in the table are based on the same cultivar. Data are shown as mean ± SD (n = 4) of four independent experiments. Different letters after the number indicated that there were significant differences among different treatments (P < 0.05). WD, well-draining, C0+WL, no CaO_2_ and waterlogging, C1+WL, 594 kg hm^-2^ of CaO_2_ in the form of base fertilizer and waterlogging, and C2+WL as in treatment C1+WL but a concentration of 864 kg hm^-2^.

**Table 3:** Effect of calcium peroxide on the accumulation of nutrients and distribution under waterlogging conditions.

Cultivars	Treatments	Stem(mg/plant)	Fruit pod(mg/plant)	Total(mg/plant)
	**N**			
XY420	WD	61.23 ± 0.82a	101.95 ± 12.38ab	163.18 ± 11.56a
	C0+WL	18.68 ± 1.80c	50.59 ± 3.38c	69.28 ± 1.61c
	C1+WL	37.73 ± 2.04b	81.09 ± 3.97b	118.82 ± 3.83b
	C2+WL	40.68 ± 2.71b	112.73 ± 6.83a	153.41 ± 8.75a
FY958	WD	110.93 ± 6.93a	143.82 ± 25.42a	254.75 ± 32.35a
	C0+WL	38.86 ± 1.30c	60.69 ± 1.58b	99.55 ± 0.28c
	C1+WL	79.89 ± 4.79b	101.84 ± 3.54ab	181.73 ± 1.26b
	C2+WL	74.79 ± 4.27b	117.25 ± 9.28a	192.04 ± 5.32b
	**P**			
XY420	WD	18.36 ± 0.64a	48.22 ± 5.17a	66.58 ± 4.26a
	C0+WL	5.92 ± 0.06c	22.03 ± 0.37b	27.95 ± 0.42c
	C1+WL	12.91 ± 0.95b	41.78 ± 6.13a	54.69 ± 6.26ab
	C2+WL	10.89 ± 0.40b	37.46 ± 3.40a	48.35 ± 3.20b
FY958	WD	31.33 ± 6.18a	48.39 ± 1.38a	79.71 ± 4.80a
	C0+WL	7.50 ± 1.21b	20.95 ± 0.50b	28.44 ± 1.70c
	C1+WL	25.56 ± 1.56a	27.56 ± 1.60b	53.11 ± 3.05b
	C2+WL	24.46 ± 0.15a	52.34 ± 7.34a	76.79 ± 7.18a
	**K**			
XY420	WD	154.16 ± 5.99a	203.71 ± 14.22a	357.87 ± 20.21a
	C0+WL	4.62 ± 0.80c	72.19 ± 1.34c	77.51 ± 1.95d
	C1+WL	24.75 ± 4.19c	114.59 ± 7.13b	139.34 ± 6.17c
	C2+WL	109.45 ± 8.49b	177.23 ± 8.06a	286.69 ± 1.55b
FY958	WD	244.37 ± 0.13a	283.04 ± 0.7a	527.41 ± 0.83a
	C0+WL	2.77 ± 0.54c	80.77 ± 10.1d	83.54 ± 10.64d
	C1+WL	61.98 ± 2.46b	136.37 ± 8.25c	198.34 ± 5.79c
	C2+WL	229.60 ± 11.05a	179.03 ± 10.93b	408.63 ± 0.13b

The standard error and significant difference analysis in the table are based on the same cultivar. Data are shown as mean ± SD (n = 4) of four independent experiments. Different letters after the number indicated that there were significant differences among different treatments (P < 0.05). WD, well-draining, C0+WL, no CaO_2_ and waterlogging, C1+WL, 594 kg hm^-2^ of CaO_2_ in the form of base fertilizer and waterlogging, and C2+WL as in treatment C1+WL but a concentration of 864 kg hm^-2^.

### Leaf senescence and resistance response to stress

**Figure 3:**
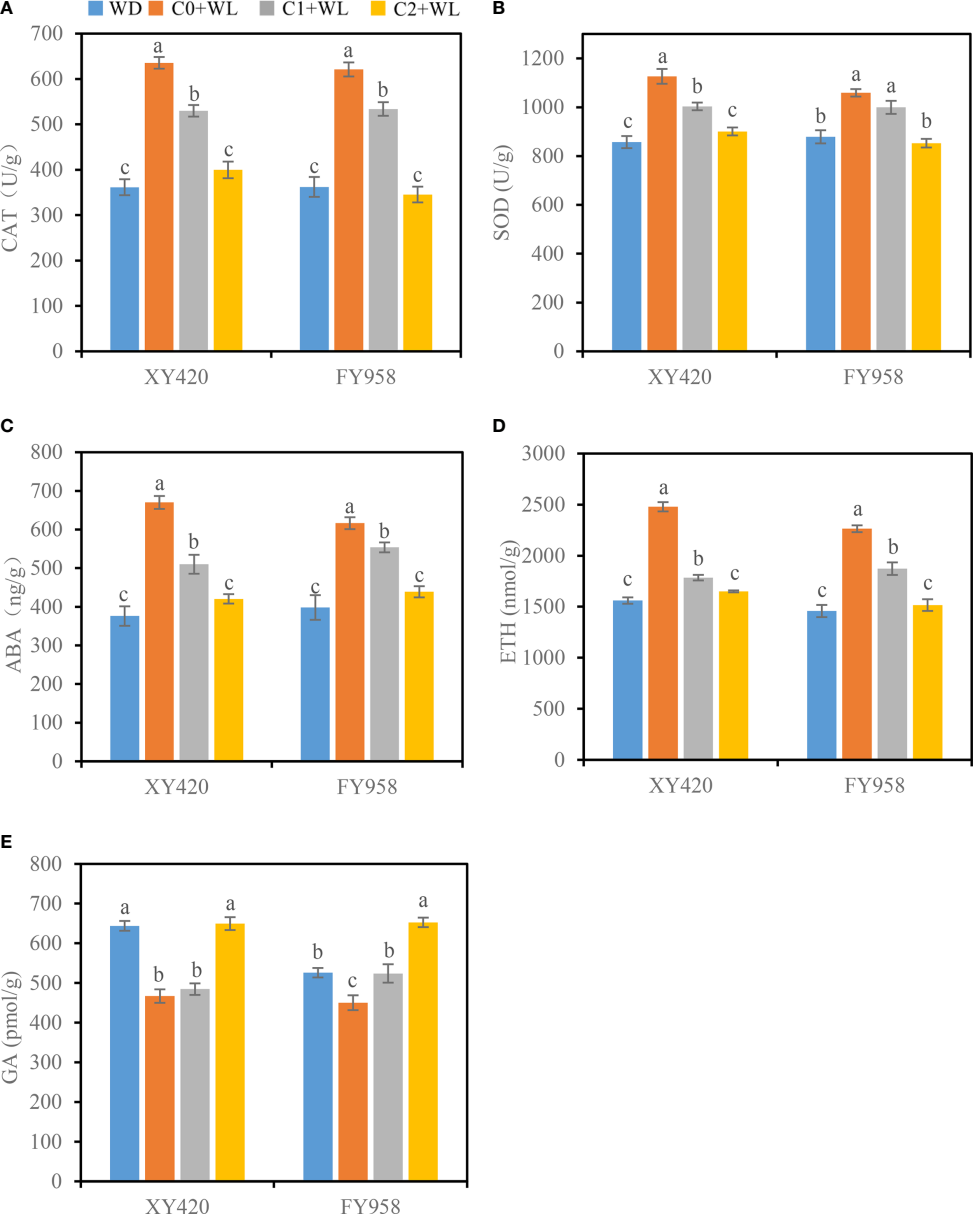
Effect of CaO_2_ on the activities of antioxidant enzymes and hormone contents in the leaves under waterlogging conditions. **(A)**, CAT, catalase; **(B)**, SOD, superoxide dismutase; **(C)**, ABA, abscisic acid; **(D)**, ETH, ethylene; **(E)**, GA, gibberellin. Error bars represent standard errors. Different letters above bars indicate significant differences between planting patterns at *P* < 0.05. WD, well-draining, C0+WL, no CaO_2_ and waterlogging, C1+WL, 594 kg hm^-2^ of CaO_2_ in the form of base fertilizer and waterlogging, and C2+WL as in treatment C1+WL but a supply of 864 kg hm^-2^.

As shown in **Figure 2C**, waterlogging stress significantly decreased the leaf SPAD values of both rape varieties, resulting in a decrease of 17.2% and 21.5% for XY420 and FY958, respectively (**Figure 2C**). Simultaneously, waterlogging stress increased the activities of CAT and SOD by 71.5%-75.9% and 20.5%-31.4%, respectively (**Figures 3A, B**). Compared with the plants subjected to waterlogging stress, the leaf SPAD values significantly improved by 35.3% and 14.7% for XY420 and FY958 in C1 and improved by 24.2% and 33.5% for XY420 and FY958 in C2. Also, CaO_2_ application decreased the activities of CAT and SOD by 15.3% and 13.3% in C1, 40.8% and 19.8% in C2, on average of these two varieties.

Many physiological and biological processes in plants are controlled by hormones. The contents of ABA and ETH in the leaves increased by 54.8%-78.2% and 55.3%-58.8% under waterlogging conditions, respectively, while the content of GA decreased by 14.3%-27.5% (**Figures 3C-E**). These conditions were improved by CaO_2_. The content of ABA in the control decreased significantly by 10.2%-37.3%, while the content of ETH decreased significantly by 17.3%-33.4%. The content of GA increased significantly by 16.4%-44.9%. The contents of hormones returned to nearly normal, particularly when treated with the high supply of CaO_2_.

### Impact on the root growth status and metabolic way

**Figure 4:**
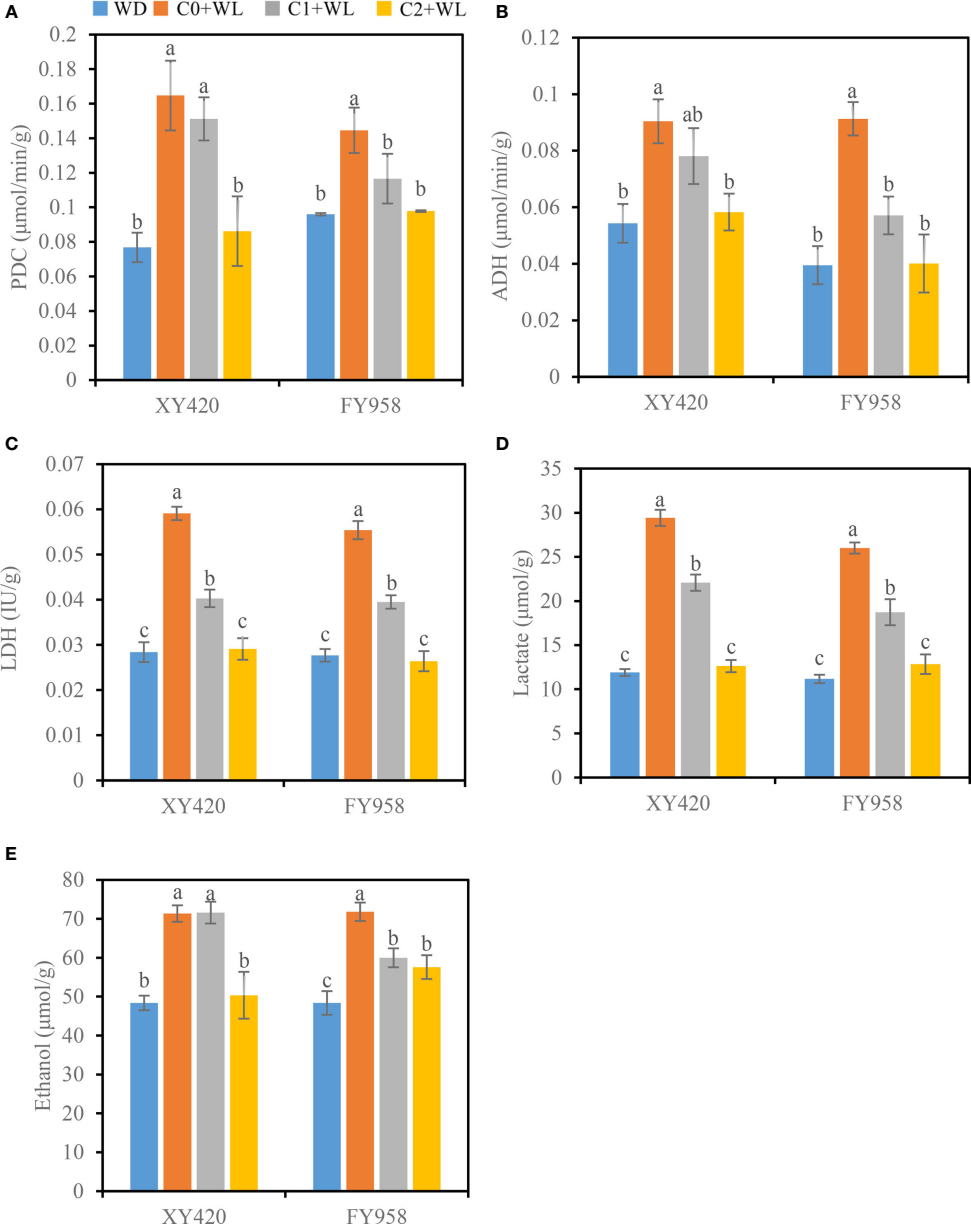
Effects of CaO_2_ on the activities of fermentative enzymes and fermentation products in roots under waterlogging conditions. **(A)**, PDC, pyruvate decarboxylase; **(B)**, ADH, alcohol dehydrogenase; **(C)**, LDH, lactate dehydrogenase; **(D)**, lactate; **(E)**, ethanol. Error bars represent standard errors. Different letters above bars indicate significant differences between planting patterns at *P* < 0.05. WD, well-draining, C0+WL, no CaO_2_ and waterlogging, C1+WL, 594 kg hm^-2^ of CaO_2_ in the form of base fertilizer and waterlogging, and C2+WL as in treatment C1+WL but a supply of 864 kg hm^-2^.

Waterlogging stress during the bud-flowering period significantly increased the activity of root fermentation enzymes in both varieties of rape (**Figure 4**). Among them, the activity of PDC increased by 50.6%-114.5%, and the activity of ADH increased by 66.5%-131.1%, while the activity of LDH increased by 50.0%-108.1%. The activities of fermentation enzymes were significantly reduced and returned to normal levels following treatment with CaO_2_. The activity of PDC decreased by 8.2% and 12.4%, that of ADH by 13.6% and 37.5%, and that of LDH by 31.8% and 28.7% in C1. And in C2 treatment, the activity of PDC decreased by 47.7% and 32.4%, that of ADH by 35.5% and 56.1%, and that of LDH by 50.8% and 52.3%. Changes in the activities of fermentation enzymes also led to changes in the accumulation of fermentation products, which increased significantly under waterlogging conditions. Lactate and ethanol increased by 143.9% and 48.4%, respectively. Treatment with CaO_2_ led to a decrease in the accumulation of fermentation products in the roots to the control level under waterlogging stress (**Figures 4D, E**). As a result, the content of lactate significantly decreased by 25.0%-57.1% and the content of ethanol by 16.5%-29.4%.

**Figure 5:**
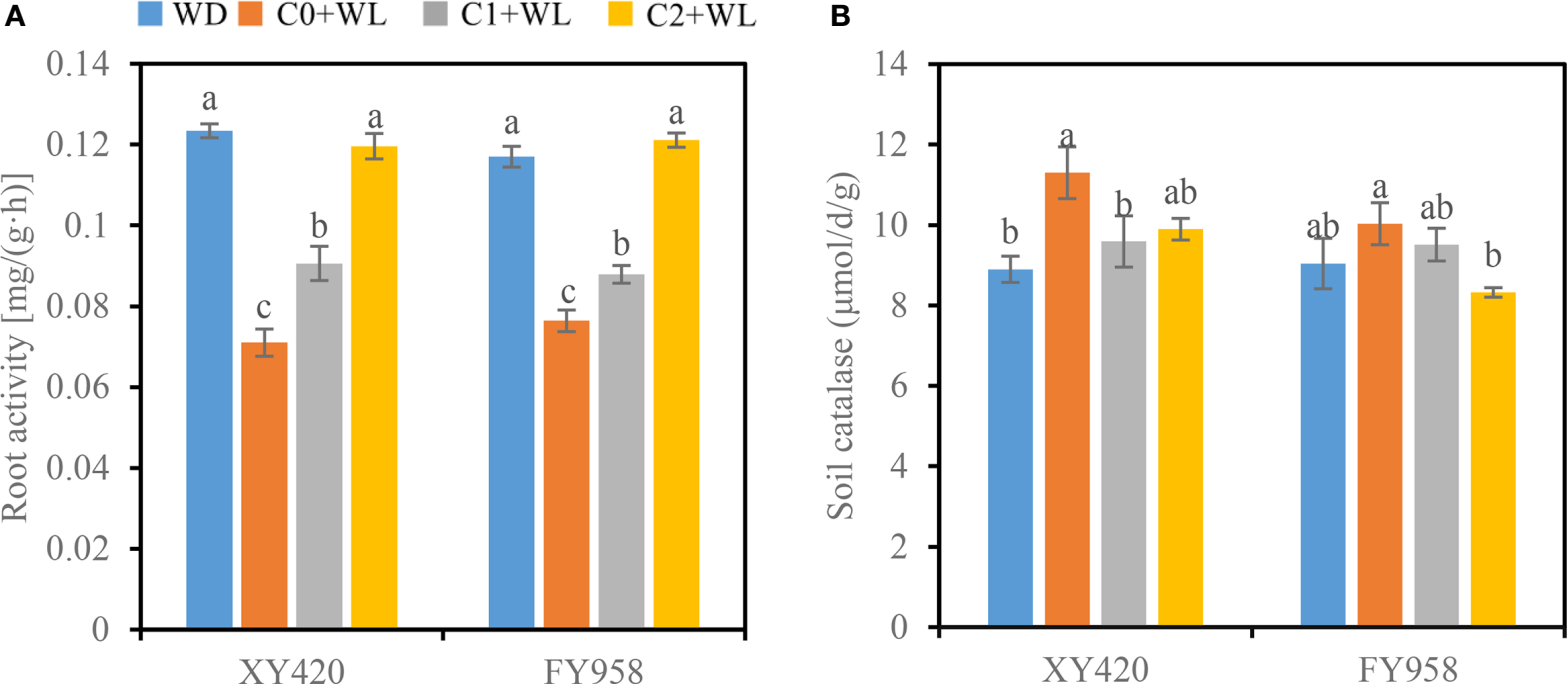
Effect of CaO_2_ on root activity and the activities of soil catalase under waterlogging conditions. Error bars represent standard errors. **(A)**, root activity; **(B)**, soil catalase. Different letters above bars indicate significant differences between planting patterns at *P* < 0.05. WD, well-draining, C0+WL, no CaO_2_ and waterlogging, C1+WL, 594 kg hm^-2^ of CaO_2_ in the form of base fertilizer and waterlogging, and C2+WL as in treatment C1+WL but a supply of 864 kg hm^-2^.

In terms of root dehydrogenase activity, waterlogging significantly reduced this activity in the roots of both rape varieties by 34.7%-42.5%. CaO_2_ can improve this condition. CaO_2_ significantly increased the root activity of waterlogged rape (**Figure 5A**). The root activity was increased by 27.6%-68.5% and 15.1%-58.5% for XY420 and FY958, respectively. High supply of CaO_2_ was more effective.

### Effects of calcium peroxide on the soil features of rape

Waterlogging for a long period promoted an increase in the activity of soil CAT, particularly for XY420, which increased by 27.0%, compared to WD treatment. The application of CaO_2_ improved this condition. The activity of soil CAT was significantly lower in the CaO_2_ soil, and decrease by 15.1% in C1, 12.4% in C2 for XY420, while reduced by 5.2% in C1, 17.0% in C2 for FY958, respectively (**Figure 5B**).

## Discussion

### Calcium peroxide alleviates the stress to plant growth and alters the antioxidant enzymes under waterlogging

Long term waterlogging stress will seriously damage plant roots, resulting in root blackening and poor development (Herzog et al., 2016). A substantial amount of this damage is irreversible (Wollmer et al., 2018; Men et al., 2020). The leaf SPAD values, the yield and the accumulation of DM of both varieties of rape were significantly reduced in waterlogged stress condition, which was largely due to the degradation of chlorophyll and damage to the photosynthetic system (Guo et al., 2004; Ploschuk et al., 2018; Tian et al., 2019). Treatment with CaO_2_ led to a substantial improvement in the leaf SPAD values and nutrients accumulation (**Table 3**), which could be attributed to the robust root system (Herzog et al., 2016; Pan et al., 2021).

Reactive oxygen species (ROS) are the normal products of plant cell metabolism. The content of intracellular ROS can also increase under waterlogging stress owing to the deficiency in oxygen (Julia and Ruth, 2005; Pucciariello et al., 2012). Hydrogen peroxide (H_2_O_2_) is another signaling molecule that plays an important role in plant stress (Steffens et al., 2011). In this study, the activities of SOD and CAT increased in waterlogging conditions (**Figure 3**) to remove ROS and H_2_O_2_ and reduce oxidative damage (Doupis et al., 2017; Hasanuzzaman et al., 2020). The activities of SOD and CAT decreased when CaO_2_ was applied, suggesting that rapeseed tended to return to normal growth. Based on the results of a previous pot experiment, calcium ion or calcium hydroxide (a products of the reaction of CaO_2_ and water) did not improve the growth status of rape under waterlogging stress (**Figure S1**), which implied that the CaO_2_ was the real work of waterlogging resistance.

Since they are signaling molecules, the plant hormones changed when the plants were subjected to waterlogging stress (Pan et al., 2021). ABA is considered a key hormone in response to waterlogging stress (Zhu, 2016; He et al., 2018). In our study, the increased ABA (**Figure 3C**) and low SPAD value in waterlogging leaves suggest that waterlogging stress probably induced early programmed senescence of rapeseed (Zhou and Lin, 1995) and up-regulated the expression of genes related to ABA synthesis (Nan et al., 2002; Zhang et al., 2016a; Zhang et al., 2016b). ABA-dependent and non-ABA-dependent signal transduction pathways are triggered to improve the resistance of plants to stress. The content of ABA in the C2+WL plants decreased to normal levels, suggesting that the plants were no longer stressed by the lack of water. Ethylene is the primary regulatory hormone that plants use to overcome waterlogging. Owing to the hypoxia stress caused by waterlogging stress, the TCA cycle of plants is limited. Therefore, alternative productivity pathways, such as the non-TCA cycle, are particularly important (Tsai et al., 2016; Bashar, 2018). Ethylene provides non-TCA cycle enzymes to maintain the survival of plants (Su et al., 2018) under waterlogging stress. Gibberellin (GA) is related to the regulation of peroxidation in plants and can antagonistically interact with ABA, which was proven to be a key hormone to improve waterlogging tolerance in rice (Ayano et al., 2015). Indeed, the application of CaO_2_ significantly increased the content of GA compared with waterlogging treatments (**Figure 3E**). The main reason was that waterlogging inhibited the synthesis and transportation of GA (Xie et al., 2003), and CaO_2_ negatively affected this inhibition.

### Calcium peroxide improves the root growth status

Waterlogging is a great threat to rape growth in a rice-rape rotation field. In this research, CaO_2_ application significantly improved the growth environment for rape roots, promoted root growth as evidenced by the higher root vitality (**Figure 5A**), and enhanced the plant’s ability to absorb nutrients from the soil (**Table 3**). These root improvements resulted in stronger plant branches and leaf SPAD value, and increased biomass compared to those under waterlogging without CaO_2_ (**Table 2**). From agronomic and physiological perspectives, vigorous root establishment and more branches formation facilitated subsequent plant growth, yield component development and final yields (Zhang et al., 2017a; Yamauchi et al., 2018; Du et al., 2021).

The pores in soil are occupied by water under waterlogging conditions, and the roots are forced to conduct anaerobic respiration owing to insufficient oxygen (Pan et al., 2021). This inefficient mode consumes a substantial amount of energy in the plant (Bailey-Serres and Voesenek, 2008). Owing to the up-regulation of anaerobic pathway, two pathways of lactic acid fermentation and alcohol fermentation were activated. ADH and PDC play key roles in the process of alcohol fermentation, and their activities are usually regarded as important indicators that reflect tolerance to plant waterlogging (Zhang et al., 2017b). Our research showed that the activities of PDC, ADH and LDH, the key enzymes involved in the glycolysis and fermentation pathway, significantly increased in waterlogging conditions (**Figure 4**). These fermentation enzymes promote the accumulation of ethanol and lactic acid in plants, which accumulate to large quantities that can be toxic (Pan et al., 2021). The increase in levels of lactate is known to result in cell acidosis, which, in turn, activates the induction of alcohol (Irfan et al., 2010).

CaO_2_ reacts with water to release oxygen, which can be used to supply the aerobic respiration of roots. The application of CaO_2_ to soil can improve the environment of rapeseed in waterlogged soil, which can be reflected in a decrease in the activities of fermentation enzymes and a reduction in the accumulation of products of these enzymes. A high supply of CaO_2_ (C2+WL) was more effective than a low supply (C1+WL), which indicated that the amount of available oxygen increased with the increase in content of CaO_2_ (Yanada, 1952; Baker and Hatton, 1987).

### Soil peroxidation decreases in response to calcium peroxide

Soil CAT is an intracellular enzyme that exists in all aerobic bacteria and most facultative anaerobic bacteria (Trasar-Cepeda et al., 2000; Shiyin et al., 2004). Catalase prevents damage to crop cells by degrading H_2_O_2_ into molecular oxygen and water (Stepniewska et al., 2009). The activity of soil CAT increased significantly under waterlogging (**Figure 5B**). This could be because the low oxygen environment in waterlogged soil promotes the proliferation of some anaerobic microorganisms (Mei et al., 2017). The effect of waterlogging on soil enzyme activity leads to changes in soil properties and negative effects on crop production (Yang et al., 2005; Gu et al., 2019; Pan et al., 2021). Relevant studies showed that the activity of catalase in waterlogged soil increased significantly with the increase in time of waterlogging, resulting in oxidation of the rape rhizosphere (Gu et al., 2019). The low oxygen environment in waterlogged soil was improved by CaO_2_, and the activity of soil CAT decreased significantly to its normal level, which suggested that the activity of soil CAT was largely affected by the supply of oxygen in the environment (Unger et al., 2009a; Unger et al., 2009b).

**Figure 6:**
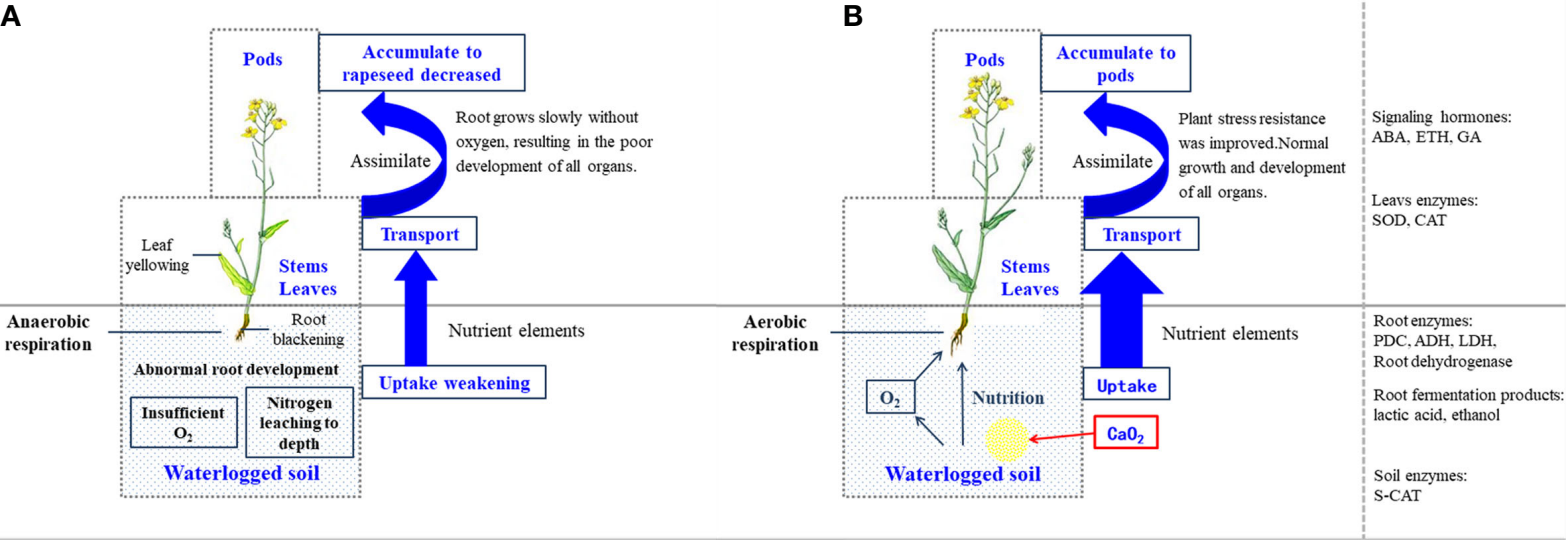
Schematic diagram of the calcium peroxide regulatory network in the regulation of rapeseed growth under waterlogging stress. **(A)** In waterlogging stress without calcium peroxide. **(B)** In waterlogging stress with calcium peroxide.

Based on the agronomic characters, amount of nutrient accumulation, and aboveground and belowground physiological responses, a potential pathway framework was constructed to reveal the functions of CaO_2_ in the regulation of rapeseed growth under waterlogging stress (**Figure 6**). A decrease in the supply of oxygen inhibits the growth of roots in waterlogging conditions. The excessive proportion of anaerobic respiration resulted in the accumulation of a large amount of lactic acid and ethanol in the root system. Damage to the root affects the growth and absorption of nutrients by the plant, which leads to a high activity of antioxidant enzymes and the release of ABA and ETH in the leaf. The plants senescence was accelerated, and the yield was reduced by affecting the number of branches and pods. The application of CaO_2_ substantially improved this negative condition. CaO_2_ reacts with soil water to supply oxygen to the roots, which enables them to maintain normal aerobic respiration. Simultaneously, the soil CAT decreased to its normal level, which helped to maintain a healthy root system. Ultimately, the yield of rapeseed and accumulation of plant nutrients were also substantially improved.

## Conclusions

The study showed that bud-flowering waterlogging stress significantly inhibited the rape growth. Under waterlogging conditions, the toxicity of fermentation products in the roots limits the absorption of nutrients and leads to an antioxidant reaction of the aboveground parts, which hinders the overall growth and development of rape. CaO_2_ application significantly improved the growth of plants and delayed the senescence of rape. Agronomic traits, such as plant height and branches, improved significantly, and the yield was increased. On the physiological level, the activities of SOD and CAT were inhibited, and the levels of ABA and ETH decreased with CaO_2_ treatments. In conclusion, 594 kg hm^-2^ ~ 864 kg hm^-2^ CaO_2_ application significantly improved the growth environment for rape roots and enhanced the plant’s ability to absorb nutrients from soil and facilitated higher yields. This study verified the feasibility of applying CaO_2_ to alleviate rape waterlogging in a rice-rape rotation field, and might have implications for agricultural adaptation under future complex climate conditions. In the future, CaO_2_ can be widely used in the form of an envelope in many dryland crops to enhance their waterlogging resistance and improve crop productivity.

## Data availability statement

The original contributions presented in the study are included in the article/**Supplementary Material**. Further inquiries can be directed to the corresponding author.

## Author contributions

ZW carried out the experiment, collected data, and with YH wrote the manuscript draft with input from all authors. SL, NJ, and CL collected and analyzed the data. LY, XR, and HS were involved in planning and supervising the work, design and implementation of the research and final revising the manuscript. All authors contributed to the interpretation of the results and editing the final manuscript. All authors contributed to the article and approved the submitted version.

## Funding

This study was supported by the National Natural Science Foundation of China (32102476), Scientific Research Foundation of Hunan Provincial Education Department (18K043), and the Double first-class construction project of Hunan Agricultural University (SYL2019042).

## Conflict of interest

The authors declare that the research was conducted in the absence of any commercial or financial relationships that could be construed as a potential conflict of interest.

## Publisher’s note

All claims expressed in this article are solely those of the authors and do not necessarily represent those of their affiliated organizations, or those of the publisher, the editors and the reviewers. Any product that may be evaluated in this article, or claim that may be made by its manufacturer, is not guaranteed or endorsed by the publisher.
