# Supplementary material for: Calcium peroxide alleviates the waterlogging stress of rapeseed by improving root growth status in a rice-rape rotation field
Source: Front Plant Sci. 2022 Nov 18;13:1048227. doi: 10.3389/fpls.2022.1048227 (PMC9718366; doi:10.3389/fpls.2022.1048227)
Supplement: Supplementary file 1 [file DataSheet_1.docx]

b

a

d

c

**Treatments**

**WD**

**CK+WL**

**CaO_2_+WL**

**CaO+WL**

**Ca(OH)_2_+WL**

**CaCO_3_+WL**

**WD**

**CK+WL**

**CaO_2_+WL**

**CaO+WL**

**Ca(OH)_2_+WL**

**CaCO_3_+WL**

Fig. S1 Effects of different calcium compounds on antioxidant enzyme activities in rape leaves and fermentative enzyme activities in roots under flooding conditions.

Note: a, SOD, superoxide dismutase; b, PDC, pyruvate decarboxylase; c, CAT, catalase; d, ADH, alcohol dehydrogenase. Data are shown as mean ± SE (n = 4) of four independent experiments. Different letters above bars indicate significant differences between planting patterns at *P* < 0.05.
